# The Greatest Problem of the Day in Dentistry

**Published:** 1915-04

**Authors:** R. Ottolengui


### THE GREATEST PROBLEM OF THE DAY IN DENTISTRY.

BY R. OTTOLENGUI, M.D.S., D.D.S.

Grieves, Hartzel and other research workers and sound thinkers in our profession, are declaring that arthritis, cardiac lesions and other dreaded diseases may often be directly traced to the septic roots of improperly filled teeth. Indeed, the dangers from septic conditions about and around the teeth have been described and printed in so many dental and medical periodicals, and evidence as to the correctness of the statements has been so convincing that no one has the temerity to deny that an improperly filled root canal may become the direct cause of invalidism and even of death.

Once the layman fully grasps this, and stops to contemplate the menace to his health that poor dentistry may engender, will it be long before he begins malpractice suits against his dentist if he goes lame in his great toe after having a front tooth filled? But how is the layman to become informed, you ask?

By his doctor! The layman accepts anything that his doctor tells him, so long as he has faith; when he loses faith he gets him another doctor and then believes what that one says. But what does the doctor know? Little enough! Altogether too little in view of the seriousness of the situation.

The following is a true narration of an actual incident, and carries a moral; two morals, in fact:

A well-known dentist called on the writer recently for an opinion as to the reading of several radiographs. The subject had an attack of rheumatism and visited her family doctor. The doctor, having read his journals, immediately suspected the teeth, and forthwith sent the woman to a radiographer, a medical radiographer. Upon receiving the films the doctor informed the woman that almost every molar and bicuspid in her mouth suffered from a blind abscess, and that until these were cured he could do nothing for her. Hence the visit of the patient to her dentist, and as a sequence his request for assistance and advice from a brother dentist.

A careful scrutiny of all the films showed first that they were not well taken, and that the depth of shadows were consequently not thoroughly reliable. Some one must have informed the doctor that a dental abscess shows dark in the films; therefore, every little dark speck in the film loomed large as an abscess to this medical mind. The first moral, then, is embodied in the rather old adage, that "a little knowledge is a dangerous thing," even for a physician. Further study of the films showed that of nine or ten suspected teeth, all but three enjoyed living pulps and sound health.

But! The other three teeth all had abscesses, and were teeth which had been filled by the dentist himself. The root fillings were imperfect, especially so since one of the root canal pastes had been used. The second and more important moral is, that dentists must learn to properly fill roots, or else very shortly be prepared to be held responsible by the patient and doctor for all manner of body ills when a radiograph can be produced that proves that his root work was imperfectly done.

But the radiographers tell us that over seventy-five per cent. of the canals of multirooted teeth, and at least fifty per cent. of single-rooted teeth, show imperfect root-canal fillings when tested with the X-ray. A number of our most careful and skillful dentists have proven that root canals can be properly treated and properly filled if the proper technique be employed, which includes at least two and often more radiographs to check up the progress and completion of the work.

With the adequate method constantly described in the literature, and with radiographic proof that his work was imperfect, what chance will the dentist have before a jury in a malpractice suit, when the medical man will testify, "This patient is crippled with arthritis for life, and the infection came from these imperfectly filled roots. Had they been correctly filled, or had they been extracted, the patient would be a well man to-day."

Of course the answer to all this is that if men do not know how to fill root canals properly, they must learn. The skill must be acquired for their own safety and for the benefit of the health of the community .

One just arrives at this deduction when some one rises on the floor in a discussion of this subject and asks this question of the assayist:

"You tell us that a tooth should better be extracted than to be left in the mouth with the root imperfectly filled. Suppose you had a practice among the poor. Suppose that a pretty young woman should come in, and you should find a dead pulp in a central incisor, not yet discolored. She tells you that five dollars is the utmost that she can spend on that tooth. Would you treat and fill the root, taking at least two radiographs, and fill the tooth likewise for that sum? Or would you extract that girl's tooth?"

A few days later a correspondent, writing of the poor fees obtainable for dental service, declares it to be his opinion that half of all the dentists fail to earn as much as three dollars per hour. Of root canal work he says: "For teeth requiring pulp removal with all treatment and final filling, five dollars is an exceptional price. Three dollars and a half is more commonly paid. I have lost a number of good families by the five dollar charge." Later on he tells us: "It is a fact that a great majority of patients are unable to pay large fees. They are supporting their families on from $800 to $1,500 a year. Mechanics earn good pay for perhaps eight months every year, no more. Clerks, small business men, saleswomen, nine-tenths of the lawyers, ministers and physicians struggle to maintain their dignity on $2,000 a year. Their family expenses keep them guessing.''

Another dentist writes: ''The use of the X-ray in root canal work is undoubtedly the best and most scientific way, but how can a dentist follow that method for patients who can not pay over five dollars for the entire service? What are you going to do for the great majority of the people? The state of affairs, as it appears to ninety per cent. of the dentists of this country, is not so much that the patient is unwilling to pay, but rather that he is unable to afford the fees for high-grade services.''

These facts and these questions bring the dental profession face to face with a grave problem. The research worker tells us that imperfect root canal fillings may produce serious systemic lesions. The radiographer tells us that his findings prove the correctness of these views, but adds that with the aid of the X-ray, roots can be properly filled and aseptic conditions maintained. Then comes the plain dentist, not the one with the rich clients, but just the plain dentist working for plain people, and he asks, ''How can I do my root fillings and get the radiographic work done, or do it myself, for the prices that my people can afford to pay? What shall I do?''

What shall we reply?—*Items of Interest* for March.

